# The impact of metal cup size on neonatal and maternal morbidity in vacuum‐assisted deliveries

**DOI:** 10.1002/ijgo.70667

**Published:** 2025-11-15

**Authors:** Matan Anteby, Tally Pinchas‐Cohen, Yoav Baruch, Anat Lavie, Sharon Maslovitz, Liran Hiersch, Yariv Yogev

**Affiliations:** ^1^ Lis Hospital for Women's Health, Tel Aviv Sorasky Medical Center, Gray Faculty of Medicine Tel Aviv University Tel Aviv Israel

**Keywords:** Malmström cup, neonatal birth trauma, operative vaginal birth, vacuum‐assisted delivery

## Abstract

**Objective:**

To evaluate whether metal cup size influences maternal and neonatal morbidity in vacuum‐assisted deliveries (VAD).

**Materials and Methods:**

Retrospective cohort at a single tertiary center (2011–2022) comparing 60‐mm versus 50‐mm Malmström cups. A 1:1 matched cohort analysis balanced maternal and obstetric variables. Primary neonatal outcomes were a birth trauma composite outcome (brachial plexus injury, cephalohematoma, subgaleal or subdural hematoma) and a severe neonatal composite outcome (neonatal intensive care unit admission, 5‐min Apgar score less than 7, umbilical artery pH less than 7.1, intraventricular hemorrhage, hypoxic ischemic encephalopathy, or neonatal convulsions). The primary maternal composite outcome included postpartum hemorrhage, third‐ or fourth‐degree perineal tears or need for postpartum blood products.

**Results:**

After matching, 1564 patients were included (782 per group). The 60‐mm cup was associated with fewer cup detachments (22 [2.8%] versus 41 [5.2%], *P* = 0.015), a shorter interval to delivery (4.3 ± 1.9 versus 4.7 ± 2.4 min, *P* = 0.003) and a comparable failure rate leading to cesarean delivery (5 [0.6%] versus 7 [0.9%], *P* = 0.56). The neonatal birth trauma composite outcome was less prevalent with 60‐mm cups (51 [6.5%] versus 72 [9.2%]; odds ratio [OR] 0.69, 95% confidence interval [CI] 0.47–0.99, *P* = 0.049) whereas the severe neonatal composite outcome did not differ (83 [10.6%] versus 96 [12.3%], *P* = 0.302). Maternal composite outcome was similar between groups; however, perineal tears were less common with 60‐mm cups (306 [39.1%] vs 369 [47.2%]; OR 0.72, 95% CI 0.59–0.88; *P* = 0.001).

**Conclusion:**

In VAD, use of a larger Malmström cup (60 mm) is associated with fewer cup detachments and a reduced risk of neonatal birth trauma without increasing maternal morbidity.

## 1 INTRODUCTION

Vacuum‐assisted deliveries (VADs) constitute approximately 3%–5% of all deliveries.^1^, ^2^ Although essential in obstetric practice, VADs are linked to potential neonatal complications such as intracranial hemorrhage, cephalohematoma, scalp lacerations, and brachial plexus injury, alongside maternal risks primarily involving perineal trauma.^3^

Vacuum cups are classified by the material used to make the cup (either soft or rigid), by the size of the cup, and by its shape, which includes primarily bell‐shaped cups and mushroom‐style cups. The Malmström cup, one of the most commonly used vacuum extraction devices, features a mushroom‐shaped metal cup made of stainless steel. It is available in diameters of 40, 50, and 60 mm.^4^

As VAD may lead to maternal and neonatal morbidity, there is a need for optimization of the VAD procedure, including the choice of the safest and most efficient cup. The clinical outcomes of different types of vacuum cups (soft versus rigid cups) have been examined in several trials and have been reviewed in a recent meta‐analysis.^5^ At present, no studies have clearly demonstrated a benefit of one type of vacuum cup over another for achieving a vaginal birth nor for preventing perinatal complications. Moreover, as far as we know, the literature is lacking data focused on comparing different metal cup sizes for adverse outcomes.

Recent work has emphasized that procedural precision and obstetric context both influence outcomes in VAD. Accurate cup placement has been linked with lower rates of failed extractions and neonatal trauma,^6^ whereas maternal complications have been associated with the type of instrument used, fetal station, duration of the second stage, and birth weight.^7^

We aimed to compare the effect of vacuum metal cup sizes on maternal and neonatal outcomes in VADs.

## 2 MATERIALS AND METHODS

### 2.1 Study design and setting

This retrospective cohort study was conducted at Lis Hospital for Women's Health, Tel Aviv Sourasky Medical Center, Israel, and included all singleton VADs between January 1, 2011, and March 31, 2023.

All women with singleton pregnancies who underwent VAD using a metallic Malmström cup were included in the study. Excluded from the study were multiple pregnancies, intrauterine fetal demises, and deliveries conducted using a non‐metallic vacuum cup or an unknown cup type.

To minimize baseline differences between groups, we performed a 1:1 matched cohort analysis. Patients in the 60‐mm group were matched with patients in the 50‐mm group according to maternal age, body mass index (calculated as weight in kilograms divided by the square of height in meters), parity, gestational age at delivery, neonatal birth weight, head circumference, use of regional analgesia, intrapartum fever, vacuum indication (non‐reassuring fetal heart rate or prolonged second stage), and fetal station. Matching was performed using exact matching for categorical variables and narrow calipers for continuous variables. After matching, 782 patients remained in each group, and unmatched patients were excluded from further analysis.

All VADs were performed by experienced staff in the presence of an attending senior physician. VAD indications consisted mainly of non‐reassuring fetal heart rate and prolonged second stage of labor. The choice of cup size was based on availability. The 60‐mm cup was introduced into practice in November 2017 and thereafter used at the physician's discretion. The vacuum device was placed when the fetal head reached a station that was below the level of the ischial spines (+1 or more in the three‐point station scale). An external force pressure of 550–600 mm Hg was used for both cup sizes. Manual protection of the perineum was routinely performed. In accordance with our institution and national guidelines, the maximum total vacuum application time was limited to 30 min and the number of cup detachments was limited to two before subsequent urgent cesarean delivery.

The primary outcomes were neonatal and maternal complications, which included maternal composite outcome (postpartum hemorrhage, third‐ or fourth‐degree perineal tears, use of postpartum blood products), neonatal birth trauma composite outcome (cephalohematoma, subgaleal hematoma, subdural hematoma, brachial plexus injury) and the severe neonatal composite outcome (need for neonatal intensive care unit admission, 5‐min Apgar score <7, umbilical artery pH <7.1, intraventricular hemorrhage, hypoxic ischemic encephalopathy, and neonatal convulsions). Secondary outcomes included time to delivery, cup detachment, and VAD failure leading to a cesarean delivery.

Demographic, clinical data were extracted from our comprehensive electronic medical record database. All patients who underwent VAD during the study period were included.

The following data were retrieved: maternal demographic and obstetric data, labor parameters, VAD indication and procedure details, and maternal and neonatal outcomes.

The study was approved by the local Institutional Review Board (IRB TLV‐0284‐08), which waived the requirement for informed consent owing to the retrospective design and use of de‐identified data.

### 2.2 Statistical analysis

The statistical analysis included several steps to ensure reliability of the findings. Initial comparisons of maternal and delivery characteristics before the decision to perform VAD revealed significant differences across multiple variables. These differences were addressed using a 1:1 matched cohort analysis. Relevant variables and their tolerance levels were entered into a matching algorithm, which paired participants with similar characteristics. Categorical variables were matched exactly and continuous variables within narrow calipers (allowable matching ranges). Participants who did not match were excluded. After matching, 782 patients remained in each group.

A power analysis was conducted before final matching to ensure adequate sample size. With a power of 80%, a significance level of 0.05, and assuming a moderate effect size based on pilot data, the required total sample size was 1172 patients (586 per group). The final matched sample of 1564 patients (782 per group) therefore exceeded the calculated requirement. Categorical variables were compared between the two study groups using χ^2^ or Fisher exact tests and are reported as frequencies and percentages. Continuous variables were compared using *t* tests or Mann–Whitney *U* tests, as appropriate. Normally distributed variables are presented as means ± standard deviation, and skewed variables as medians with interquartile ranges. All statistical tests were two‐sided, with *P* values less than 0.05 considered statistically significant. Analyses were performed in SPSS version 26 (IBM, Armonk, NY, USA).

## 3 RESULTS

**FIGURE 1:**
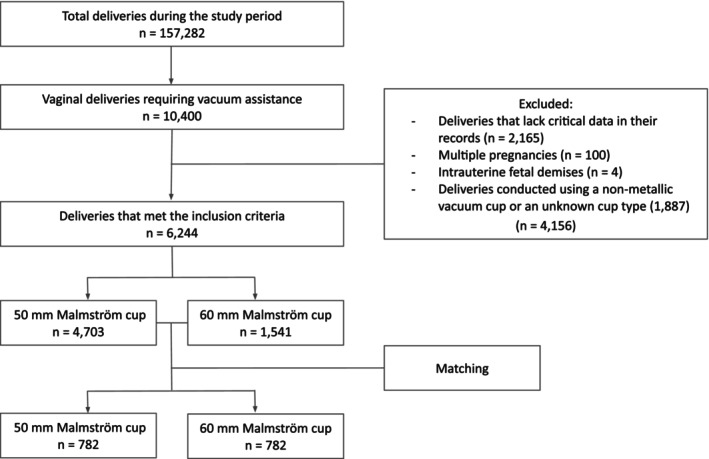
Study design and patient selection flowchart.

During the study period, 157 282 deliveries took place in our center, of them, 10 400 (6.6%) were delivered by VAD, out of which, 6244 (60%) met the inclusion criteria. Overall, 4703 (75.3%) were conducted using a 50‐mm Malmström cup and 1541 (24.7%) with the 60‐mm Malmström cup (Figure 1). Background demographic, obstetric and labor characteristics of the cohort are presented in Table 1. To address baseline differences, we applied 1:1 matched cohort analysis. After matching for maternal and fetal characteristics as well as the pre‐VAD parameters mentioned above, in each group 782 comparable cases were enrolled. Baseline characteristics are presented in Table 1.

**TABLE 1:** Background characteristics—vacuum‐assisted delivery matched cohort.^a^

Characteristics	60‐mm cup (*n* = 782)	50‐mm cup (*n* = 782)	*P* value^b^
Maternal demographic and obstetric data			
Age, years	31.5 ± 3.8	31.5 ± 3.7	0.937
BMI	22 ± 2.9	22 ± 2.9	0.978
Nulliparity	726 (92.8%)	726 (92.8%)	0.539
Previous vacuum assisted delivery	6 (0.8%)	3 (0.4%)	0.253
Previous cesarean delivery	20 (2.6%)	23 (2.9%)	0.379
IVF pregnancy	76 (9.7%)	74 (9.5%)	0.466
Labor parameters			
Gestational age, week	40.1 ± 1	40.1 ± 1	0.985
Head circumference at birth, cm	34.9 ± 1.3	34.9 ± 1.2	0.47
Birth weight, g	3319 ± 362	3296 ± 349	0.21
Length of second stage, h:min	02:14 ± 1:00	02:16 ± 1:10	0.512
Intrapartum fever	35 (4.5%)	29 (3.7%)	0.262
Meconium‐stained amniotic fluid	273 (34.9%)	272 (34.8%)	0.5
Pre‐eclampsia	12 (1.5%)	9 (1.2%)	0.331
Regional anesthesia	765 (97.8%)	765 (97.8%)	0.569
Episiotomy	711 (90.9%)	711 (90.9%)	>0.99
Station of descent			
S + 3	0 (0.0%)	0 (0.0%)	0.535
S + 2	710 (90.8%)	710 (90.8%)	
S + 1	72 (9.2%)	72 (9.2%)	
Position			
Occipito‐anterior	668 (85.4%)	668 (85.4%)	0.529
Occipito‐posterior	114 (14.6%)	114 (14.6%)	
Vacuum indication			
NRFHR	725 (92.7%)	725 (92.7%)	0.539
Prolonged second stage of labor	478 (61.1%)	478 (61.1%)	0.521

Abbreviations: BMI, body mass index (calculated as weight in kilograms divided by the square of height in meters); IVF, in vitro fertilization; NRFHR, non‐reassuring fetal heart rate.

^a^ Data are given as mean ± standard deviation or numbers (percentage).

^b^ Continuous variables were compared using Student *t*‐test or Mann–Whitney *U*‐test, as appropriate. Categorical variables were compared using χ^2^ or Fisher exact test.

The use of the 60‐mm cup was associated with a statistically significant reduction in the mean interval from VAD initiation to delivery (4.31 ± 1.9 versus 4.65 ± 2.4 min, mean difference −0.34 min, 95% confidence interval [CI] –0.56 to −0.12, *P* = 0.003). The use of a 60‐mm cup was also associated with a reduced risk of cup detachment (2.8% versus 5.2%, odds ratio [OR] 0.52, 95% CI 0.31–0.89, *P* = 0.015). The rate of failed VAD necessitating cesarean delivery was comparable in both groups (0.6% versus 0.9%, OR 0.71, 95% CI 0.22–2.25, *P* = 0.56) (Table 2).

**TABLE 2:** Delivery outcomes—matched cohort.^a^

	60‐mm cup (*n* = 782)	50‐mm cup (*n* = 782)	*P* value^b^	OR (95% CI)
Procedure aspects				
Vacuum length, min	4.31 (1.9)	4.65 (2.4)	*0.003*	
Cup detachment	22 (2.8%)	41 (5.2%)	*0.015*	0.52 (0.31–0.89)
Vacuum failure	5 (0.6%)	7 (0.9%)	0.562	0.71 (0.22–2.25)
Maternal outcome				
Maternal composite outcome^c^	84 (10.7%)	75 (9.6%)	0.451	1.14 (0.82–1.58)
Perineal tears	306 (39.1%)	369 (47.2%)	*0.001*	0.72 (0.59–0.88)
Third‐ or fourth‐degree tear	9 (1.2%)	10 (1.3%)	0.817	0.9 (0.36–2.22)
Postpartum hemorrhage	76 (9.7%)	67 (8.6%)	0.43	1.15 (0.81–1.62)
Use of blood products	30 (3.8%)	28 (3.6%)	0.789	1.07 (0.64–1.81)
Perinatal outcome				
Neonatal birth trauma composite outcome^c^	51 (6.5%)	72 (9.2%)	*0.049*	0.69 (0.47–0.99)
Brachial plexus injury	3 (0.4%)	1 (0.1%)	0.625	3.01 (0.31–29)
Cephalohematoma	29 (3.7%)	44 (5.6%)	0.072	0.65 (0.4–1.04)
Subgaleal hematoma	20 (2.6%)	25 (3.2%)	0.449	0.8 (0.44–1.44)
Subdural hematoma	2 (0.3%)	5 (0.6%)	0.452	0.4 (0.08–2.06)
Severe neonatal composite outcome^c^	83 (10.6%)	96 (12.3%)	0.302	0.85 (0.62–1.16)
Umbilical artery pH <7.1	57 (9.2%)	57 (12.4%)	0.093	0.72 (0.49–1.06)
5‐min Apgar score <7	5 (0.6%)	8 (1%)	0.403	0.62 (0.2–1.91)
Neonatal intensive care unit admission	31 (4.0%)	36 (4.6%)	0.532	0.86 (0.52–1.4)
Intraventricular hemorrhage	1 (0.1%)	1 (0.1%)	>0.99	1 (0.06–16)
Hypoxic ischemic encephalopathy	6 (0.8%)	15 (1.9%)	*0.048*	0.39 (0.15–1.02)
Convulsions	5 (0.6%)	10 (1.3%)	0.195	0.5 (0.17–1.46)

Abbreviations: CI, confidence interval; OR, odds ratio.

^a^ Data are given as median (interquartile range) or numbers (percentage).

^b^ Continuous variables were compared using Student *t*‐test or Mann–Whitney *U*‐test, as appropriate. Categorical variables were compared using χ^2^ or Fisher exact test.

^c^ Composite outcomes: Maternal composite outcome: postpartum hemorrhage, third‐ or fourth‐degree perineal tear, use of blood products; Neonatal birth trauma composite outcome: cephalohematoma, subgaleal hematoma, subdural hematoma, brachial plexus injury; Severe neonatal composite outcome: neonatal intensive care unit, 5‐min Apgar <7, umbilical artery pH <7.1, intraventricular hematoma, hypoxic ischemic encephalopathy, convulsions.

Rates of maternal composite outcome did not differ between the groups (OR 1.14, 95% CI 0.82–1.58, *P* = 0.451). The risk for perineal tears was lower in the 60‐mm cup group compared with the 50‐mm cup group (39.1% versus 47.2%, OR 0.72, 95% CI 0.59–0.88, *P* = 0.001).

The rate of neonatal birth trauma composite outcome was lower with the 60‐mm cup (OR 0.69, 95% CI 0.47–0.99, *P* = 0.049). The severe neonatal composite outcome did not differ significantly between the groups (OR 0.85, 95% CI 0.62–1.16, *P* = 0.3), but there was a trend toward fewer cases of hypoxic ischemic encephalopathy with the 60‐mm cup (OR 0.39, 95% Cl 0.15–1.02, *P* = 0.048).

## 4 DISCUSSION

In this study, we aimed to investigate the effect of vacuum cup size on maternal and neonatal outcomes in VADs. Our main findings were as follows. (1) The use of a larger 60‐mm Malmström cup was associated with a reduced cup detachment and a statistically shorter interval to delivery compared with the 50‐mm cup. (2) The risk of neonatal birth trauma was higher in the 50‐mm group. (3) The maternal composite outcome was similar between the groups.

Previous research has primarily compared the performance of soft versus rigid vacuum cups, with the general conclusion being that rigid cups result in fewer delivery failures but more fetal trauma, but show no significant difference in maternal trauma compared with soft cups.^5^ To our knowledge, this study represents the first clinical study aiming to assess the influence of vacuum cup size on these outcomes, by comparing two different diameters of the metallic Malmström vacuum cup. Huang et al.^8^ investigated the biomechanical effects of different sizes of silicone rubber vacuum extractors on the fetal head through a finite element analysis, concluding that vacuum extractors with larger diameters led to greater reaction force, stress, and strain on fetal heads.

These findings describe theoretical mechanical load in a simulated model, but our data on metallic cups, demonstrating reduced neonatal trauma with the larger 60‐mm cup, likely reflect improved adhesion and fewer detachments, which offset the potential for increased localized stress.

Furthermore, there was no corresponding increase in maternal adverse outcomes with the larger cup. The superior performance of the 60‐mm cup in terms of neonatal birth trauma was reflected in the shorter mean time from VAD initiation to delivery and fewer detachments, although cesarean delivery rates were not significantly affected. Previous studies have linked prolonged procedures and higher rates of device detachments to worse neonatal outcomes.^9^, ^10^, ^11^, ^12^, ^13^ However, while statistically significant, the 21‐s difference in vacuum timing between the two cup sizes is unlikely to be of clinical significance.

Our observation linking the use of the 50‐mm diameter cup to a more challenging vacuum extraction may indicate the mechanism through which fetal injury occurs: the use of a smaller cup may result in an inappropriate exertion of force on the fetal scalp, leading to detachment and thereby contributing to fetal injury. A possible contributing factor to the complexity of the VAD with a smaller diameter cup could be the presence of significant caput succedaneum. It is known that the formation of caput succedaneum before cup application may adversely affect the tractive force available for extraction.^14^ Unfortunately, due to the absence of data on caput succedaneum rates in our study, we were unable to evaluate its correlation with the procedure outcomes in each group.

When assessing each neonatal outcome individually, one could notice that the main difference highlighted by the composite outcome lies in the incidence of cephalohematomas, which, although important, are typically not linked with serious morbidity. Nevertheless, this trend is also reflected in our study in the rates of more serious conditions such as subgaleal hematoma, hypoxic ischemic encephalopathy, and convulsions, yet not reaching statistical significance.

In terms of maternal outcomes, our study demonstrated that the use of the 60‐mm cup did not worsen the primary maternal composite outcome and was even associated with a reduction in second‐degree perineal tears, after matching for episiotomy rates.

No significant differences were observed in the risk of third‐ or fourth‐degree tear or postpartum hemorrhage. The correlation between a smaller cup size and a lower rate of perineal tears may be explained by the doubled rate of cup detachments and significantly longer VAD time, yet we would expect a similar rise in the additional maternal parameters.

The results of this study open opportunities for further investigation into the optimal selection of vacuum cup size in clinical practice. Future research should aim to explore additional factors that might influence the outcomes of VADs, such as variations in fetal factors, maternal anatomy, and labor characteristics. Furthermore, studies that include data on caput succedaneum and its impact on the effectiveness of VAD could provide additional insights into why smaller cups might be less effective and more traumatic. Investigating whether similar results hold in different clinical settings, such as lower‐volume centers, could shed light on the generalizability of these findings.

In the absence of explicit guidelines for selecting the appropriate size of vacuum extraction cup based on labor or maternal parameters, the choice of cup relies on the practitioner's preference. It is reasonable to assume that user preference takes into consideration relevant labor parameters, such as fetal weight, head circumference, head station and position, and other factors. To minimize the influence of these parameters on delivery outcomes, it was crucial to conduct a thorough matching analysis, which minimized the impact of confounding factors. A potential limitation of our study is selection bias, as larger cups are often chosen for more technically challenging extractions. This bias could have influenced our findings; however, as these cases would be expected to have higher complication rates, the persistence of improved outcomes with the 60‐mm cup likely reflects a true and possibly conservative effect.

It should be noted that due to inaccurate and missing data on estimated fetal weight and head circumference before performing the procedure, we used birth weight and neonatal head circumference. As these are accurate measurements, they may still differ from what the performing physician anticipated beforehand, possibly affecting the choice of cup size in a manner unknown to us. Another limitation in our study is related to its generalizability. The investigated institution is a central tertiary center that performs approximately 12 000 deliveries annually, of which 6%–7% are VADs. Forceps‐assisted deliveries are not routinely performed. As a result, the practitioners exhibit a high specialization in VADs. This may account for the relatively short duration of the vacuum procedures in our study and the relatively low failure rate within our institution, in contrast to a much higher rate for soft and rigid cups, as was reported in a recent meta‐analysis.^5^

In conclusion, the use of a larger Malmström metal cup (60‐mm diameter) during VAD is associated with a decreased risk of neonatal birth trauma without an increase in maternal adverse outcomes, compared with the 50‐mm diameter device. This finding may influence physician's choice of cup size during VADs with subsequent reduction of procedure‐related complications.

## AUTHOR CONTRIBUTIONS

MA and YY contributed to the conceptualization and visualization; MA and LH contributed to the methodology; MA, TP, and YB contributed to the formal analysis; MA, TP, YB, AL, and SM contributed to the investigation; MA and TP contributed to the data curation; MA wrote the original draft; and YY supervised the study. All authors contributed to the writing (review and editing) and they all meet ICMJE criteria and approved the final manuscript.

## FUNDING INFORMATION

This research received no external funding. No commercial organization provided supplies, services, or financial support for this study.

## CONFLICT OF INTEREST STATEMENT

The authors have no conflicts of interest.

## Data Availability

De‐identified data that support the findings of this study are available from the corresponding author upon request.
